# Hashimoto’s Encephalopathy: Clinical Features, Therapeutic Strategies, and Rehabilitation Approaches

**DOI:** 10.3390/biomedicines13030726

**Published:** 2025-03-17

**Authors:** Nicola Manocchio, Valerio Massimo Magro, Livio Massaro, Andrea Sorbino, Concetta Ljoka, Calogero Foti

**Affiliations:** Physical and Rehabilitation Medicine, Tor Vergata University, 00133 Rome, Italy; livio.massaro@students.uniroma2.eu (L.M.); andrea.sorbino@ptvonline.it (A.S.); concetta.ljoka@ptvonline.it (C.L.); foti@med.uniroma2.it (C.F.)

**Keywords:** steroid-responsive encephalopathy associated with autoimmune thyroiditis, Hashimoto’s encephalopathy, thyroid microsomal antibodies, autoimmune diseases of the nervous system, rehabilitation

## Abstract

Hashimoto’s encephalopathy (HE), also known as steroid-responsive encephalopathy associated with autoimmune thyroiditis (SREAT), is an autoimmune disorder with heterogeneous presentation that poses diagnostic challenges. This review synthesizes the current literature to clarify the clinical, laboratory, and radiological features of SREAT/HE, including the diagnostic utility of thyroid peroxidase (TPO) antibodies, cerebrospinal fluid (CSF) abnormalities, and neuroimaging findings. Cognitive impairment and behavioral changes are common in HE, but specific manifestations vary widely, which can lead to misdiagnosis. While elevated TPO antibodies are frequently observed, a direct causal relationship with HE is unlikely, and their presence may indicate a general state of autoimmunity. Corticosteroids remain the cornerstone of treatment, although responses vary, and alternative immunosuppressive agents or intravenous immunoglobulin may be needed in some cases. Evidence regarding rehabilitation for people affected by HE is limited, but neurorehabilitation strategies adapted from other neurological conditions, including cognitive re-education (CR), physical therapy, and psychosocial support, may be beneficial. Further research is needed to elucidate the underlying mechanisms of SREAT, refine the diagnostic criteria, and develop more targeted and effective therapies, including rehabilitation strategies, for this debilitating neurological disorder.

## 1. Introduction

Hashimoto’s encephalopathy (HE), first described by Brain et al. and also known as steroid-responsive encephalopathy associated with autoimmune thyroiditis (SREAT), is an autoimmune disorder. Its heterogeneous presentation requires multidisciplinary investigation and multifaceted evaluation, making precise nosographic and pathogenetic definition challenging [1]. Neurological manifestations can occur in several systemic diseases, sometimes even preceding the primary pathology. Consequently, physiatrists, internists, and neurologists are frequently consulted to evaluate patients with acute or subacute encephalopathy and its associated outcomes [2,3,4,5]. The differential diagnosis for encephalopathy is broad (Table 1); however, clinical features and findings from blood tests, cerebrospinal fluid (CSF) analysis [6,7,8], electroencephalography (EEG) [9,10,11], and neuroimaging often, but not always, lead to an accurate diagnosis [12,13,14]. From a clinical and internal medicine perspective, the initial step typically involves excluding infectious etiologies, followed by consideration of autoimmune or inflammatory processes [15,16,17,18,19]. Suspicion for these etiologies may arise based on inflammatory and autoimmune markers in serum and CSF, as well as meningeal and parenchymal abnormalities observed on brain magnetic resonance imaging (MRI) [20].

Autoimmune encephalopathies encompass various forms, including paraneoplastic [21,22,23] and idiopathic limbic encephalitis [24,25], which are typically well-defined by characteristic serological and neuroimaging abnormalities. Furthermore, idiopathic autoimmune encephalopathy is often defined by a clinical response to steroids [26,27,28,29,30]. However, the pathogenesis of HE remains debated and under investigation. Thyroid peroxidase (TPO) antibodies, organ-specific autoantibodies and serological indicators for autoimmune thyroiditis (Hashimoto’s thyroiditis), are frequently elevated in patients with SREAT [31,32]. Nevertheless, their presence may indicate a general state of autoimmunity in neurological disorders with autoimmune pathogenesis rather than specifically indicating HE [33,34,35]. A direct causal relationship between TPO and HE is in fact considered unlikely [36]. Thus, significant uncertainties persist regarding this condition, including the spectrum of clinical and laboratory manifestations, associated radiological findings, the clinical significance of TPO titers, and the typical outcomes of steroid treatment. The current literature lacks sufficient data to address these challenges, hindering both the diagnosis and the management of this disorder.

This narrative review aims to synthesize the existing literature to clarify the clinical, laboratory, and radiological features of SREAT. The review will explore the diagnostic utility of TPO, CSF abnormalities, and neuroimaging findings while addressing the variability in treatment response to corticosteroids. Furthermore, it will investigate potential rehabilitation approaches for individuals affected by HE. By analyzing these aspects across diverse studies, this work seeks to improve the recognition and diagnostic accuracy of SREAT and provide insights into its management. Ultimately, the review aims to stimulate further research into the underlying mechanisms of SREAT and its differentiation from other forms of autoimmune encephalitis

## 2. Clinical and Neurological Features

Cognitive impairment and behavioral changes are defining features common to all forms of encephalopathy, including HE. Boelen et al. observed that these characteristics are present in both adult and pediatric populations [37].

There have been reports of HE being unrecognized, with patients misdiagnosed as mentally disturbed [38,39,40]. Singh et al. presented a case of a 55-year-old male who presented predominantly with psychiatric symptoms and rapidly progressing dementia and was treated with high doses of intravenous steroids and risperidone [41]. In another case, a 33-year-old male with psychotic symptoms, also treated with steroids, was diagnosed with HE; treatment of this patient led to normalization of the anti-thyroglobulin antibody titer, which had been elevated before treatment [42]. Another case report described a 31-year-old man with psychiatric symptoms characterized by delusions, disorganized speech, talkativeness, obsessive thoughts, acute episodes of memory loss, a reduced need for sleep, and irritability for approximately 4 months; this patient was also treated with steroids, with good results and the regression of the psychiatric symptoms [43]. Amamou et al. described a case characterized by hypochondriacal symptoms associated with high levels of anti-thyroid peroxidase antibodies; in this case, steroid treatment improved the psychiatric clinical symptoms, allowing the reduction of risperidone until its discontinuation [44].

Reports have identified other clinical features in young patients, such as seizures, tremor, ataxia and cerebellar signs, and dystonia [45,46]. In addition to a four-case report [47], a recent case series by Goenka et al. confirmed the presence of variably associated symptoms and the typical age of diagnosis, between 8 and 18 years, in pediatric patients with HE [48].

The clinical presentation of HE, characterized by memory disorders and progressive cognitive deterioration leading to dementia, has been well-documented in the scientific literature [1]. Characteristically, memory changes in HE are not acute at onset; rather, they manifest as subtle, slowly progressive disorders that may be initially overlooked. This insidious progression can make early diagnosis challenging. Katagiri N et al. recently described a case of a 69-year-old man with Hashimoto’s encephalopathy presenting with the subacute progression of amnesia, highlighting the importance of considering HE in older adults with unexplained cognitive decline [49]. A retrospective study of 204 patients with encephalopathy found cognitive deficits to be the most frequent clinical presentation, affecting over 70% of those diagnosed with HE [50]. Aladdin Y et al. described a case of a non-elderly adult patient whose initial symptom was a progressive decline in recent memory, eventually impairing basic daily activities [51]. Similarly, John R et al. reported comparable memory loss in a patient of the same age, noting that cognitive impairment is a hallmark of HE, with symptoms including confusion, hallucinations, and impairment of the sleep–wake cycle [52]. Epileptic status is a well-documented manifestation of Hashimoto’s encephalopathy, often reported as sporadic cases in both younger and older patients [53,54]. In one Italian clinical experience, a previously healthy 19-year-old woman presented with generalized tonic–clonic seizures; plasmapheresis led to improvements in both her clinical condition and electroencephalogram, along with a decrease in anti-thyroid antibody titers [55]. Other cases describe decreased levels of consciousness with tonic–clonic jerks and hearing crises associated with antithyroid antibodies [56,57]. Refractory status epilepticus in a 14-year-old boy prompted diagnostic suspicion in another instance [58]. Ercoli et al. studied 31 patients with HE and status epilepticus, reporting generalized convulsive status epilepticus in 52%, non-convulsive status epilepticus in 29%, focal status epilepticus in 4%, generalized absence status epilepticus in 3%, and epilepsia partialis continua in 3% of cases [59,60]. Alink J et al. suggest measuring anti-microsomal antibodies in all children with unexplained seizures, hallucinations, or confusion and initiating prednisolone treatment if positive [61]. Similarly, Osman et al. advocated for this approach in a 60-year-old patient hospitalized for seizures of unknown origin [62]. Several mechanisms may contribute to the genesis of epilepsy in HE, with autoimmunity playing a potentially significant role. Thyroid-associated antibodies such as TPO, anti-thyroglobulin antibodies, thyrotropin receptor antibodies, and NEA antibody targets for cortical neurons and endothelial cells have been identified in patients with HE with epilepsy [63]. However, the precise role of these antibodies in epileptic pathogenesis remains unclear. Research suggests that cerebral hypoperfusion and edema-induced brain dysfunction due to autoimmune-mediated vasculitis may contribute to seizure disorders [64]. HE should be considered in the differential diagnosis for patients presenting with seizures of unknown etiology. Other characteristic features of SREAT include an oscillatory symptom course [65], tremor, myoclonus, transient aphasia, sleep abnormalities, seizures, and gait difficulties [66]. Additionally, some studies have reported migraine, neuropsychiatric features, and lateralized sensory and motor deficits [67,68].

Given the above, the hallmark presenting feature of HE is a non-specific encephalopathy characterized by an alteration of mental status and consciousness ranging from confusion to coma and impaired cognitive function (Table 2).

## 3. Laboratory Abnormalities

Several laboratory abnormalities associated with HE emerged from the literature analysis, reflecting HE’s autoimmune nature. These include positivity for anti-thyroperoxidase antibodies [69,70], anti-thyroid microsome antibodies [71], and anti-thyroglobulin antibodies [68]. Furthermore, HE may present with positivity for anti-nuclear antibodies and anti-extractable nuclear antigen antibodies, as well as rheumatoid factor [72,73]. Additional findings can include positivity for anti-gliadin antibodies, elevated erythrocyte sedimentation rates, elevated C-reactive protein, and elevated serum aminotransferase levels [74] (Table 3).

From the analyzed papers, it emerged that thyroid antibody levels in SREAT are variable, and the severity of encephalopathy does not correlate with antibody titers. The key clinical finding is the presence, not the level, of serum thyroid antibodies, suggesting SREAT should be considered, even with mildly elevated levels, in patients with encephalopathy. SREAT should also be considered regardless of thyroid status (euthyroid or mildly hypothyroid) [68,69,70,71]. Elevated serum levels of liver aminotransferases are also frequently observed. It remains unclear whether these elevated liver enzymes indicate autoimmune hepatitis or another process, warranting further investigation [74]. More specific antibodies related to the nervous system, such as those against gangliosides or the brain enzyme enolase, have also been investigated. Patients with anti-NH2-terminal alpha-enolase antibodies tend to exhibit acute encephalopathy, particularly as studied in Japan, where Hashimoto’s disease is highly prevalent. Japanese researchers in the early 2000s found a high prevalence of these antibodies in patients with SREAT [63]. Japanese researchers have reported that the serum anti-NH2-terminal of α-enolase (NAE) antibodies exhibit a specificity of 91% for SREAT, with a sensitivity of 50% [75]. Matsunaga et al. analyzed the serum of 84 patients and concluded that anti-NAE antibodies are a valuable diagnostic marker for HE [76]. This is supported by Yoneda’s findings, which emphasize the high specificity of anti-NAE antibodies for HE [77]. This suggests that anti-NAE antibodies could be a useful diagnostic marker for HE. Further research is needed to fully elucidate the role of anti-NAE antibodies in the diagnosis and pathogenesis of HE and related disorders.

## 4. Abnormalities in Cerebrospinal Fluid Analysis

Elevated CSF protein levels are frequently reported in patients with HE, but normal CSF protein levels, the absence of lymphocytic pleocytosis (elevated white blood cell count >4/μL), normal IgG synthesis rates, and the absence of oligoclonal bands do not exclude the diagnosis of SREAT [78] (Table 4).

As already stated, Hashimoto’s thyroiditis, characterized by altered levels of anti-thyroid peroxidase and/or anti-thyroglobulin antibodies in CSF or blood, can be associated with HE [1,79]. While these antibodies are valuable diagnostic markers, they may not be directly pathogenic, as they can also be detected in healthy individuals, indicating that thyroiditis and encephalopathy represent nonspecific, but distinct, events of an aggressive immune system; however, they could serve as markers of the treatment response [80]. A multicenter retrospective study conducted between 2014 and 2020 revealed that misdiagnoses of autoimmune encephalitis are common [14]. Insidious onset, positivity for non-specific serum antibodies, and the incorrect application of diagnostic criteria contribute to frequent misdiagnosis. Such misdiagnoses can lead to morbidity due to unnecessary immunotherapies and the delayed initiation of appropriate treatment [81]. Studies have also demonstrated the limited specificity of not only TPO antibodies but also NEA antibodies in diagnosing HE [82]. Examination of CSF may reveal the presence of AMPAR2 (α-amino-3-hydroxy-5-methyl-4-isoxazole-propionic acid receptor) antibodies, suggesting a potential pathogenic role in HE, given the importance of this glutamate receptor in synaptic transmission, memory, and learning [83]. The overinterpretation of non-specific serum antibodies seems a major contributor to misdiagnosis. Therefore, in addition to blood tests, examination of CSF characteristics appears essential for accurate diagnosis.

In addition to their diagnostic utility, CSF parameters can serve as markers of treatment response; for instance, Gliebus et al. monitored IgG levels in CSF, alongside the mini-mental state examination (MMSE), to assess treatment efficacy [84].

The non-specific nature of serum antibodies for HE diagnosis presents considerable challenges and poses a potential threat for misdiagnosis.

## 5. Abnormalities in Radiological Investigations

The MRI abnormalities found in review were consistent with an active encephalopathic process, including diffuse white matter signal abnormalities and meningeal enhancement (Table 5).

In several patients, these abnormalities were resolved after steroid therapy, which is an interesting phenomenon. The majority of patients diagnosed with SREAT had normal neuroimaging findings, including normal cerebral angiography findings [85]. These imaging features are different from the prominent mesial temporal lobe (MTL) abnormalities encountered in subacute inflammatory or autoimmune encephalitis or paraneoplastic limbic encephalitis [86]. One case even mimicked a tumor-like lesion located at the level of the caudate nucleus [87]. In one case of a 37-year-old patient with depressive symptoms, MRI revealed symmetric and bilateral areas of abnormally high signals on T2-weighted and fluid-attenuated inversion recovery (FLAIR) images involving the mesial temporal lobes, caudate nuclei, and putamina. Interestingly, these abnormalities did not disappear after steroid treatment [88]. In a recent case described by Ide T et al., a patient in his late 60s was characterized by FLAIR magnetic resonance imaging (MRI) that showed white matter hyperintensities that had not been apparent before admission. These brain MRI abnormalities were associated with an autoimmune antibody pattern characterized by the various abnormalities described in the laboratory abnormalities section—serum anti-thyroglobulin and anti-thyroid per oxidase antibody titers were elevated. Furthermore, anti-NH2-terminal α-enolase antibodies were positive. Again, the abnormalities found on MRI did not change after treatment [89]. However, MRI is not always indicative, and sometimes this test can be negative. In a case described by Ogbebor O et al., studied for delirium in an elderly patient (84 years old), MRI imaging of the brain, cerebrospinal fluid analysis, and paraneoplastic screening were all negative, and the only noteworthy factors were the high titer of anti-thyroperoxidase antibodies and the marked clinical response to steroid treatment [90]. Therefore, like the previous characteristics, this one alone, whether it is pathological or shows nothing, is still not sufficient to formulate a diagnosis of SREAT. MRI is among the most widely used neuroimaging tools for diagnosing encephalitis, as it can be crucial in both diagnosis and in ruling out clinical mimics of encephalitis. In fact, volumetric and functional MRI is used to define the outcomes of encephalitis and can describe the cellular and functional reorganization at the brain level during the acute phase of the disease [13]. A study conducted on 30 patients with HE, followed between January 2010 and April 2017, analyzed imaging via MRI and functional imaging; routine MRI revealed negative results in 8 cases and abnormal findings in 22 cases. Among the 22 abnormal cases, 9 showed small cerebral vascular disease, and 13 had non-specific abnormalities (12 mainly in supratentorial white matter, 11 had multiple lesions, and 2 presented with lesions complicated by cerebellar atrophy). The lesions were focal or confluent, punctate or small patchy, and displayed abnormal signal intensity, characterized by iso- or hypo-intensity on T1-weighted imaging and hyperintensity on both T2-weighted imaging and fluid-attenuated inversion recovery. Moreover, 12 out of 15 lesions showed no enhancement. In seven cases with abnormalities on DWI, three sudden acute cases showed hyperintensity on DWI and hypo-intensity on the apparent diffusion coefficient, while four sub-acute or slow-onset cases showed hyperintensity on DWI and increased apparent diffusion coefficient values. Additionally, three cases showed localized intracranial artery stenosis, and in two cases, magnetic resonance spectroscopy indicated a significantly lower N-acetylaspartate peak, a higher choline peak, and a visible lactate or lipid peak. Follow-up data from seven cases showed no change in three cases, while four cases exhibited changes including softening lesions (2/4), remitted and relapsed lesions (1/4), and the rapid progression of brain atrophy with a negative initial MRI finding (1/4). The authors concluded that routine MRI combined with functional imaging can reveal different features of HE, with routine MRI showing multifocal or confluent lesions in the white matter, mostly without enhancement; functional imaging can reveal pathological characteristics of different phases of acute or chronic ischemia and demyelinating changes of HE [91]. However, it should be considered that in 50% of cases, MRI may appear negative. HE can present with T2/FLAIR hyperintensities in the MTL regions, mimicking side effects, and cause memory deficits, seizures, and psychiatric symptoms. There is a particular “migratory pattern”, meaning the appearance of signal anomalies in new sites while others disappear. In the white matter, we find leukoencephalopathy with confluent T2/FLAIR changes [92], while functional imaging shows the pathological features of acute and chronic ischemia and the effects of demyelination due to HE [91]. The migratory pattern and leukoencephalopathy can help in the differential diagnosis between SREAT and autoimmune encephalitis (AE) [92]. In conclusion, the role of the radiologist in this disease, as in other types of encephalitis, is crucial for initiating the diagnosis. Thanks to the radiologist, the patient can be directed toward a complete diagnostic process. This is essential because, for a better therapeutic response and to detect potential malignancies, an early diagnosis represents a favorable factor [14].

## 6. Pharmacological Treatment

Pharmacological treatments for Hashimoto’s encephalopathy primarily focus on immunosuppression to mitigate the presumed autoimmune-mediated neurological damage, with corticosteroids serving as the cornerstone of treatment [80] (Table 6).

High-dose corticosteroids, such as intravenous methylprednisolone followed by oral prednisone, are frequently employed to suppress the inflammatory cascade and reduce antibody-mediated neurotoxicity [93]. Fiore et al. emphasize that the profound improvement in mental status following steroid administration is a hallmark of HE, reinforcing its classification as a steroid-responsive encephalopathy [74]. The initiation of corticosteroid therapy is critical, especially in patients presenting with severe symptoms (e.g., altered consciousness and myoclonus), as it can help differentiate HE from other encephalopathies [94]. However, the clinical response to corticosteroids can vary significantly among patients, with some experiencing dramatic improvement while others exhibit a more gradual or incomplete response, necessitating careful monitoring and individualized treatment adjustments [95]. In cases where patients exhibit an inadequate response to corticosteroids or experience unacceptable side effects, alternative immunosuppressive agents, such as azathioprine, methotrexate, or cyclophosphamide, may be considered as steroid-sparing agents. The use of these agents requires careful consideration of their potential toxicities and the need for regular monitoring of hematological and hepatic function [96].

Intravenous immunoglobulin represents another immunomodulatory therapy that has demonstrated efficacy in some patients with Hashimoto’s encephalopathy, particularly those who are refractory to corticosteroids or other immunosuppressants. The mechanism of action of intravenous immunoglobulin in Hashimoto’s encephalopathy is not fully understood but may involve the blockade of receptors, modulation of complement activation, and neutralization of pathogenic antibodies [97]. Given the potential for long-term neurological sequelae and the impact on the QoL, early and aggressive intervention is warranted to optimize outcomes and minimize disability in individuals affected by this challenging condition. In addition, thyroid hormone levels should be monitored and normalized, as thyroid dysfunction can exacerbate neurological symptoms [98]. Additional research is needed to fully elucidate the underlying mechanisms of Hashimoto’s encephalopathy and to develop more targeted and effective therapies for this debilitating neurological disorder.

## 7. Rehabilitation for People with HE

Evidence regarding rehabilitation for people affected by HE is limited. A PubMed search using the terms “Hashimoto’s Encephalopathy” AND “Rehabilitation” yielded only six results (consulted on 20 February 2025), none of which presented direct rehabilitation data. A similar search of Scopus produced the same outcome. It is thus necessary to abstract from other conditions and consider how rehabilitation for people with neurological conditions can be applied in HE.

Rehabilitation for people with neurological conditions encompasses a wide range of therapeutic approaches aimed at enhancing recovery and improving the QoL for individuals affected by various neurological disorders [99,100,101]. The complexity of these conditions necessitates a multifaceted approach that integrates physical therapy, occupational therapy, and emerging technologies.

The role of physical therapy in neurorehabilitation is pivotal. High-intensity neurorehabilitation has been shown to benefit both younger and older patients, suggesting that age should not be a barrier to receiving appropriate rehabilitation services [102]. Techniques focusing on promoting neuroplasticity, such as task-oriented training and constraint-induced movement therapy, have been developed to enhance motor learning and functional recovery in patients with hemiparesis [103]. Cognitive re-education (CR) is a specialized therapeutic approach aimed at improving cognitive functions that may be impaired due to neurological conditions. CR focuses on enhancing cognitive abilities such as memory, attention, executive function, and problem-solving skills, which are crucial for daily living and the overall quality of life [104]. One of the key aspects of CR is its adaptability to various neurological conditions. A systematic review highlighted CR effectiveness specifically in Parkinson’s disease, where cognitive training was shown to positively impact several cognitive domains, including attention and executive functions [105].

Considering the multifaceted impact of Hashimoto’s encephalopathy, neurorehabilitation necessitates a tailored approach that addresses the cognitive and physical impairments resulting from the disease. A comprehensive rehabilitation strategy should integrate CR, physical therapy, and psychosocial support to optimize patient outcomes. CR plays a crucial role in addressing the cognitive impairments associated with Hashimoto’s encephalopathy. Given that patients often experience progressive cognitive decline, interventions should focus on enhancing cognitive functions such as memory, attention, and executive functioning. Evidence suggests that CR techniques, including computer-assisted cognitive training, can be beneficial for improving cognitive outcomes, although specific studies on Hashimoto’s encephalopathy are limited [106]. This approach can be particularly effective when combined with emotional regulation strategies, which are essential for managing the psychiatric symptoms often seen in Hashimoto’s encephalopathy [107]. In addition to CR, physical therapy is essential for enhancing functional outcomes. It aids in reducing motor disabilities and improving overall physical functioning by fostering neuroplasticity and promoting functional independence [108]. Moreover, education can play a pivotal role in the rehabilitation process of several conditions, and HE is no exception. Education empowers patients and caregivers to actively participate in the rehabilitation process, monitor for recurrence or progression of the disease, and make informed decisions about treatment options [109,110].

Lastly, a multidisciplinary approach to neurorehabilitation is essential for addressing the complex needs of patients with HE. Collaboration among physiatrists, neurologists, physiotherapists, psychologists, and other healthcare professionals can facilitate comprehensive care that addresses both the neurological and psychosocial aspects of the condition [111].

## 8. Future Directions

Autoimmune encephalitis is increasingly recognized; however, this increased awareness has paradoxically led to more frequent misdiagnoses and the inappropriate application of diagnostic criteria, particularly in antibody-negative cases. Misdiagnoses typically stem from the following: failure to adhere to established clinical diagnostic requirements for AE; inadequate evaluation of inflammatory changes on MRI and CSF; and limited analysis of brain tissue, often relying on techniques that assess few antigens. While the data presented may improve the recognition of SREAT, its underlying pathogenesis remains unclear. Given the nonspecific nature of laboratory and neurological findings, coupled with an age of onset spanning several decades, it is not surprising that alternative initial diagnoses are frequently considered. The role of thyroid autoimmunity in HE pathogenesis is further complicated by the fact that elevated serum anti-TPO antibody levels are present in approximately 10% of healthy adults and that this prevalence increases with age. While antibodies against the thyroid are commonly assessed, antibodies directed against enolase may represent a more specific marker and potential pathogenic factor. Future studies should focus on better characterizing the pathological titers and function of anti-enolase antibodies in this condition. To improve diagnostic accuracy, we recommend adopting the flow diagram and study population by thyroid antibody levels from the retrospective study of Dumrikarnlert et al. [50] and applying the 2016 revised criteria by Graus et al. [16] (Table 7). These criteria incorporate clinical history, examination, CSF, MRI findings, and EEG results to assess the likelihood of an autoimmune cause and have demonstrated high specificity in clinical practice. Although many questions remain, we hope that this review will enhance the recognition of patients with potential autoimmune or inflammatory mechanisms underlying their encephalopathy and stimulate further research into SREAT and other forms of non-vasculitic autoimmune inflammatory encephalitis. A broad differential diagnosis should be considered, and misdiagnosis occurs in many settings, including at specialized centers.

## 9. Conclusions

This narrative review aimed to synthesize the current literature to clarify the clinical, laboratory, and radiological features of SREAT/HE. Despite its initial description several decades ago, SREAT remains a challenging diagnosis due to its heterogeneous presentation and the uncertainties surrounding its pathogenesis. Cognitive impairment and behavioral changes are common, but the specific manifestations can vary widely, sometimes leading to misdiagnosis. While elevated TPO antibodies are frequently observed, their direct causal relationship with HE is unlikely, and their presence may merely indicate a general state of autoimmunity.

SREAT could be considered as a potential diagnosis in patients presenting with unexplained encephalopathy, especially when accompanied by elevated thyroid antibodies. However, diagnosis should not rely solely on TPO titers, as these are not pathognomonic for HE and do not correlate with the severity of the condition. CSF analysis and neuroimaging, particularly MRI, can aid in diagnosis, although findings are often nonspecific or normal. Misdiagnosis of autoimmune encephalitis is common, and clinicians should be aware of the limitations of non-specific serum antibodies.

Corticosteroids remain the cornerstone of treatment, though responses can vary, necessitating individualized treatment adjustments. Inadequate responses to corticosteroids may warrant the use of alternative immunosuppressive agents or intravenous immunoglobulin. Furthermore, the normalization of thyroid hormone levels seems crucial.

Considering that thyroid autoantibodies are highly prevalent, increasing with age from 10% to 30%, it is unsurprising that many patients with encephalopathies also exhibit thyroid autoantibody positivity. However, “steroid responsiveness” in autoimmune encephalopathies remains the most critical diagnostic criterion and is entirely unrelated to thyroid dysfunction. Consequently, the term steroid-responsive autoimmune encephalopathies (SRAE) may represent a more accurate and descriptive diagnostic label, helping to eliminate confusion caused by the misleading association with thyroid autoimmunity. It is essential to highlight that correlations between thyroid autoantibodies and encephalopathy do not establish causality.

Evidence regarding rehabilitation for people affected by HE is limited, but neurorehabilitation strategies, including CR, physical therapy, and psychosocial support, adapted from other neurological conditions, may be beneficial. A multidisciplinary approach is essential to address the complex needs of patients with HE. Further research is needed to fully elucidate the underlying mechanisms of SREAT, refine diagnostic criteria, and develop more targeted and effective therapies, including rehabilitation strategies, for this debilitating neurological disorder.

## Figures and Tables

**Table 1:** Most frequent clinical conditions to consider in the differential diagnosis of SREAT.

Condition	Key Features/Distinguishing Factors
**Infectious Etiologies**	Encephalitis (e.g., viral) must be ruled out. May present with fever and other signs/symptoms of infection. Sepsis-associated encephalopathy should also be considered.
**Stroke/TIA**	Interruption of blood flow to the brain leading to neurological deficits. TIA causes similar symptoms that resolve quickly.
**Autoimmune/Inflammatory Processes**	SREAT itself is an autoimmune-mediated inflammatory disease. Vasculitis may be present. Rapid progression of neuropsychiatric symptoms.
**Psychiatric Disorders**	Can mimic encephalopathy with altered mental status and behavioral changes. SREAT can present with acute psychiatric symptoms.
**Metabolic Encephalopathy**	Encephalopathy due to metabolic derangements. Hepatic, uremic, or respiratory encephalopathy should be considered.
**Drug Toxicity/Withdrawal**	Overdosing of certain medications. Withdrawal of sedatives or opioids, alcohol withdrawal delirium.
**Creutzfeldt–Jakob Disease (CJD)**	SREAT can mimic sporadic CJD.

**Table 2:** HE manifestations.

Clinical Domain	Symptomatology
**Cognitive/Behavioral**	Behavioral abnormalities, cognitive abnormalities, dementia
**Motor**	Tremor (shaking), ataxic gait, lateralized motor deficits, myoclonus
**Speech**	Transient aphasia
**Seizures**	Generalized seizures, partial seizures, status epilepticus
**Sleep**	Hypersomnia, insomnia
**Sensory**	Headache, lateralized sensory deficits
**Psychiatric**	Psychosis, paranoia, hallucinations, delusions, mood disturbance

**Table 3:** Laboratory abnormalities of Hashimoto’s encephalopathy.

Antibody/Marker	Description
**Anti-Thyroperoxidase (TPO) Antibodies**	Elevated levels of antibodies against thyroid peroxidase, a key enzyme in thyroid hormone production.
**Anti-Thyroid Microsome Antibodies**	Elevated levels of antibodies against thyroid microsomes, which are cellular components involved in thyroid hormone synthesis.
**Anti-Thyroglobulin Antibodies (Ab anti-Tg)**	Elevated levels of antibodies against thyroglobulin, a protein used by the thyroid gland to produce thyroid hormones. The presence of anti-thyroglobulin antibodies should not be taken as the definitive diagnostic criteria since these antibodies could be associated with other autoimmune encephalopathies, which include anti-LGI1, anti-NMDA, and anti-Caspr2.
**Anti-Nuclear Antibodies (ANA)**	Presence of antibodies that target the cell nucleus, indicating a possible autoimmune process.
**Anti-Extractable Nuclear Antigen (ENA) Antibodies**	Presence of antibodies against extractable nuclear antigens, which are specific proteins within the cell nucleus.
**Rheumatoid Factor**	Elevated levels of rheumatoid factor, an antibody associated with rheumatoid arthritis and other autoimmune conditions.
**Anti-Gliadin Antibodies**	Presence of antibodies against gliadin, a component of gluten, which may indicate gluten sensitivity or celiac disease.
**Erythrocyte Sedimentation Rate (ESR)**	Elevated ESR, a non-specific marker of inflammation in the body.
**C-Reactive Protein (CRP)**	Elevated CRP, another non-specific marker of inflammation in the body.
**Serum Aminotransferase Levels (AST/ALT)**	Elevated levels of AST and ALT, liver enzymes that may indicate liver inflammation or damage.
**Thyroid Functional Status**	Heterogeneous thyroid function, including euthyroid (normal thyroid function), subclinical hypothyroid (mildly underactive thyroid), and subclinical hyperthyroid (mildly overactive thyroid).
**Anti-Amino (NH2)-Terminal of α-Enolase Antibodies**	Presence of antibodies against the amino-terminal of alpha-enolase, a glycolytic enzyme found in various tissues, including the brain.

**Table 4:** CSF abnormalities in HE.

CSF Parameter	Abnormality
**Protein Level**	Elevated
**Leukocyte Count**	Elevated (>4 leukocytes/μL)
**IgG Synthesis Rate**	Elevated
**IgG Index**	Elevated
**Oligoclonal Bands**	Increased (>2)

**Table 5:** Abnormalities in radiological investigations in SREAT.

Feature	Description
**MRI Findings**	Normal or nonspecific.
**White Matter Abnormalities**	Diffuse white matter signal abnormalities (hyperintensities on T2/FLAIR). Can be focal or confluent, punctate or small patchy. Leukoencephalopathy with confluent T2/FLAIR changes can be present.
**Meningeal Enhancement**	May be present.
**Temporal Lobe Involvement**	T2/FLAIR hyperintensities in the mesial temporal lobe (MTL) regions.
**Basal Ganglia/Thalamic Involvement**	Can be involved. One case mimicked a tumor-like lesion in the caudate nucleus. Symmetric and bilateral high signals on T2-weighted and FLAIR images involving the caudate nuclei and putamina.
**Brainstem Involvement**	Can be involved.
**DWI Abnormalities**	Hyperintensity on DWI with corresponding hypo-intensity on ADC in acute cases; hyperintensity on DWI with increased ADC in subacute/slow onset cases.
**Intracranial Artery Stenosis**	Localized intracranial artery stenosis.
**Functional Imaging (MRS)**	Decreased N-acetylaspartate (NAA) peak, increased choline peak, visible lactate or lipid peak.
**Conus Medullaris Involvement**	Can be present.
**Migratory Pattern**	Appearance of signal anomalies in new sites while others disappear.
**Response to Steroid Therapy**	Some abnormalities may resolve after steroid therapy. However, some abnormalities may not change after treatment.

**Table 6:** Pharmacological treatments for SREAT.

Treatment	Description	Dosage/Administration	Considerations
**Corticosteroids**	Cornerstone of treatment; suppresses the inflammatory cascade and reduces antibody-mediated neurotoxicity. A profound improvement in mental status following steroid administration is a hallmark of HE.	Initial treatment: IV methylprednisolone (e.g., 500–1000 mg/day for 3–5 days), followed by oral prednisolone (1–2 mg/kg/day), with gradual tapering.	Clinical response can vary; monitor carefully and adjust treatment individually. Long-term high doses associated with significant side effects. Doses may need to be reduced to reduce the incidence of iatrogenic Cushing’s syndrome and the risk of osteoporosis.
**Immunosuppressive Agents**	Alternative agents for patients with inadequate response to corticosteroids or unacceptable side effects. Examples include azathioprine, methotrexate, or cyclophosphamide.	Dosage varies depending on the agent.	Requires careful consideration of potential toxicities and regular monitoring of hematological and hepatic function. Used as steroid-sparing agents.
**Intravenous Immunoglobulin (IVIG)**	Immunomodulatory therapy effective in some patients, especially those refractory to corticosteroids or other immunosuppressants. The mechanism may involve the blockade of receptors, modulation of complement activation, and neutralization of pathogenic antibodies.	Dosage: IVIg 2 gm/kg was given twice at 1-month intervals. Number of plasma exchange courses may vary from 3 to 10.	Monitor for side effects.
**Rituximab**	Anti-B-cell therapy (monoclonal antibody directed against CD20).	Dosage: 1000 mg intravenously on days 1 and 14, and then once every 6 to 9 months depending on the white blood cell count; 375 mg/m², twice at 1-week intervals.	Can be of value in steroid-refractory cases.Well-tolerated and induces a sustained remission without the need for additional corticosteroids. Monitor carefully for side effects due to low levels of immunoglobulins.
**Plasmapheresis**	A method for removing unwanted substances (toxins, metabolic substances, autoantibodies) from the blood.	During plasmapheresis, blood is removed from the affected individual and blood cells are separated from plasma. The plasma is then replaced with other human plasma, and the blood is transfused back into the affected individual. Number of plasma exchange courses may vary from 3 to 10.	Thought to induce clinical improvement by removing other antibodies, autoimmune complexes, cytokines, and/or other inflammatory mediators currently unknown.
**Levetiracetam**	Suggested due to its anti-inflammatory properties in treating associated epileptic syndrome.	Not specified in the provided search results.	Indicated in patients with seizure-like epileptic syndromes.
**Thyroid Hormone Management**	Monitoring and normalization of thyroid hormone levels.	As needed to maintain euthyroid status.	Thyroid dysfunction can exacerbate neurological symptoms. Consider levothyroxine or antithyroid drugs.

**Table 7:** 2016 Graus et al. revised diagnostic criteria for HE [16].

Criterion	Description
**1. Encephalopathy**	Presence of encephalopathy with one or more of the following: seizures, myoclonus, hallucinations, or stroke-like episodes.
**2. Thyroid Disease**	Evidence of thyroid disease, typically subclinical or mild overt hypothyroidism.
**3. Brain MRI Findings**	Brain MRI is either normal or shows non-specific abnormalities.
**4. Thyroid Antibodies**	Presence of serum thyroid antibodies, specifically anti-thyroid peroxidase (TPO) and/or anti-thyroglobulin antibodies.
**5. Exclusion of Other Neuronal Antibodies**	Absence of other neuronal antibodies in both serum and cerebrospinal fluid (CSF).
**6. Exclusion of Alternative Causes of Encephalopathy**	Reasonable exclusion of alternative causes of encephalopathy. This requires a thorough evaluation to rule out other potential etiologies before attributing the symptoms to Hashimoto’s encephalopathy. It is therefore important to rule out infectious and non-infectious etiologies.

## Data Availability

All data used in this review are available in the text.
